# A Rare Case of P‐Null Phenotype Identified During Preoperative Screening

**DOI:** 10.1002/ccr3.72456

**Published:** 2026-04-29

**Authors:** Alireza Abdollahi, Aysan Nozheh, Hassan Sohrabinia, Samaneh Salarvand

**Affiliations:** ^1^ Department of Pathology, Imam Khomeini Hospital Complex Tehran University of Medical Sciences Tehran Iran; ^2^ BA in Medical Laboratory, Central Laboratory of Imam Khomeini Hospital Complex Tehran University of Medical Sciences Tehran Iran

**Keywords:** anti‐PP1Pk, immunohematology, P‐null phenotype, rare blood group, transfusion management

## Abstract

A 46‐year‐old woman scheduled for an elective total abdominal hysterectomy was incidentally found to have a rare P‐null (p) phenotype during routine preoperative testing. Although her ABO/Rh type was A positive, the antibody screen demonstrated strong pan‐reactivity with all panel cells, while the autocontrol was negative, indicating an alloantibody. This prompted referral to a reference immunohematology laboratory, where the pan‐agglutinating antibody was ultimately identified as specific anti‐P1Pk, consistent with the patient's rare P‐null phenotype. Consequently, the patient was flagged as requiring future transfusions with P‐null, ABO/Rh‐compatible red blood cells. Surgery proceeded without the need for transfusion, but identification of this rare phenotype has substantial implications for long‐term care, particularly given the known association of anti‐PP1Pk with severe hemolytic transfusion reactions and recurrent pregnancy loss. This case highlights the importance of comprehensive pretransfusion testing, early collaboration with specialized laboratories, meticulous documentation, and proactive transfusion planning, particularly in regions where such phenotypes may be underrecognized.

## Key Clinical Message

Pan‐agglutinating alloantibodies should raise suspicion for antibodies against high‐prevalence antigens. In this case, an initially unidentified pan‐agglutinating alloantibody was ultimately shown to be anti‐P1PK in a patient with the rare P‐null phenotype, highlighting the importance of reference laboratory support in transfusion management. Early recognition prevents severe hemolytic reactions, especially in under‐recognized regions.

## 1 Introduction

The P1PK blood group system encompasses a set of high‐frequency antigens—P, P1, and Pk—that are present in nearly all individuals, making antibodies directed against them both uncommon and clinically significant. The P‐null (p) phenotype, characterized by the complete absence of these antigens, is exceptionally rare worldwide. Individuals with this phenotype frequently develop naturally occurring anti‐PP1Pk, a potent antibody capable of causing severe hemolytic transfusion reactions and complications in pregnancy [1, 2]. Because these antibodies target antigens found on almost all donor red blood cells, their detection poses substantial challenges for transfusion services, often requiring coordination with rare‐donor registries and specialized immunohematology laboratories [3, 4].

Despite its clinical importance, the P‐null phenotype is often identified incidentally, most commonly during preoperative or prenatal screening. Awareness of this rare condition is limited, particularly in regions where few cases have been documented, which increases the risk of delayed recognition or inappropriate transfusion [5, 6]. The present case highlights the discovery of a P‐null phenotype in a patient undergoing routine preoperative evaluation, underscoring the crucial role of standardized antibody screening and timely referral for advanced testing. By presenting this case, we aim to reinforce the importance of early identification, careful transfusion planning, and multidisciplinary management in patients with rare blood group phenotypes.

## 2 Case History/Examination

A 46‐year‐old woman presented to the gynecology service for management of symptomatic uterine prolapse. She was admitted for elective surgical treatment. The patient was married, born in Tehran, and had one child. She reported no history of miscarriage, blood transfusion, or prior hematologic disorders. Her past medical history was otherwise unremarkable, and she was scheduled for a total abdominal hysterectomy (TAH), which was performed several days after admission without intraoperative complications.

Preoperative laboratory testing revealed her blood group as A positive. Routine antibody screening using a minipanel showed uniform 2+ reactivity with all three screening cells at room temperature, in the albumin and AHG phases, while the autocontrol and DAT were negative. Antisera and albumin were supplied by Immunodiagnostic Co. (Germany). All tests were carried out using the tube method according to standard protocols. The antibody screening test employed IMMUCOR screening reagent cells (USA) as per the manufacturer's instructions. Quality control checks were performed on every antiserum and screening vial, all of which produced satisfactory results. The patient's blood bank test results are summarized in Table 1.

**TABLE 1:** Patient antibody screening test results.

Test cell	RT	Alb	AHG
Cell 1	2+	2+	2+
Cell 2	2+	2+	2+
Cell 3	2+	2+	2+

This pattern raised suspicion for an antibody directed against a high‐prevalence red cell antigen. To clarify the findings, her sample was referred to the Iranian Blood Transfusion Organization (IBTO) Pathobiology Laboratory for specialized immune‐hematologic evaluation.

## 3 Differential Diagnosis

Anti‐PP1Pk (P‐null phenotype), technical interference.

## 4 Conclusion and Results (Outcome and Follow‐Up)

At the IBTO, antibody identification was carried out using an 11‐cell panel, and the reactions were read and interpreted in all three phases according to the manufacturer's protocol. Phenotyping of the patients' red blood cells for RhCE and K antigens was performed, and the results showed no evidence of antigen deficiency. Due to a suspicious anti‐PP1Pk antibody, crossmatching of the patients' sera with a confirmed P‐null donor was performed. The National Reference Laboratory at the Institut National de la Transfusion Sanguine (INTS), Paris, confirmed the donor rare p phenotype, also known as Tj(a‐), and the presence of potent anti‐PP1Pk antibodies. No reaction was observed in this crossmatch, whereas all crossmatches with other blood units showed positive results.

Antibody identification result: Antibody to a high‐prevalence antigen, with laboratory findings supporting anti‐PP1Pk antibodies.

Recommendation: For transfusion support, the patient would require ABO‐ and Rh‐compatible P‐null red blood cells, crossmatched at the AHG phase.

These results suggested that the patient possesses the rare P‐null phenotype, characterized by the absence of P, P1, and Pk antigens. No immediate transfusion was required perioperatively; however, identification of this rare phenotype carried major implications for future transfusion management, necessitating prior coordination with reference laboratories and rare donor registries.

## 5 Discussion

The present case highlights the incidental identification of a rare P‐null phenotype with probable anti‐PP1Pk in a 46‐year‐old woman undergoing elective gynecologic surgery. Routine preoperative testing showed an A Rh(D)‐positive blood group with a positive antibody screen against a high‐prevalence antigen, and reference laboratory workup suggested anti‐PP1Pk with a recommendation to use only ABO/Rh‐compatible P‐null red cells if transfusion were required. Although no transfusion was ultimately needed, this finding has major implications for her future surgical and obstetric care.

Most individuals express P and P1, while P‐null individuals completely lack all three P1PK antigens because of loss‐of‐function variants in the A4GALT gene [7]. The p phenotype is extremely rare, with an estimated prevalence of approximately 5.8 per million in certain populations [3]. Recent molecular characterizations from Asia, particularly India, have expanded the known allelic spectrum. Studies have identified novel and rare variants in the α‐1,4‐galactosyltransferase (A4GALT) gene, including a novel allele resulting in the p phenotype in an Indian blood donor and other pathogenic variants in Indian patients, underscoring the genetic heterogeneity underlying this condition in regional populations [8].

Because P1PK antigens are almost universally expressed, individuals with the P‐null phenotype typically develop a naturally occurring alloantibody directed against the high‐frequency PP1Pk antigen complex, historically known as anti‐Tja (anti‐PP1Pk) [9].

Anti‐PP1Pk is usually a mixture of IgG and IgM and is strongly complement‐activating. It is capable of causing severe acute hemolytic transfusion reactions even with small amounts of incompatible red cells and has also been implicated in hemolytic disease of the fetus and newborn (HDFN) and early recurrent miscarriage [9].

Several recent case reports and small series illustrate the breadth of clinical problems associated with the P‐null phenotype. Joshi et al. described a woman with a P‐null phenotype in India who suffered a severe hemolytic transfusion reaction when the least‐incompatible blood was transfused; molecular analysis showed a 26‐bp deletion in A4GALT (A4GALT*01 N.019) [10]. Other reports from India and elsewhere have documented trauma patients and surgical candidates in whom anti‐PP1Pk was discovered during urgent cross‐matching or after unexplained hemolysis [1, 4, 7]. These reports, along with the recent molecular findings from the region, highlight the ongoing importance of serologic vigilance and the growing role of genetic confirmation in managing this rare phenotype [11].

In contrast, our patient was identified before any exposure to donor blood, and no transfusion was ultimately needed during her TAH. This favorable course underscores the value of routine preoperative antibody screening and appropriate escalation to a reference laboratory when a pan‐reactive pattern against screening cells suggests an antibody to a high‐prevalence antigen. Although molecular genotyping was not performed for the patient due to logistical limitations, serologic compatibility with red cell units previously characterized by molecular methods supported the suspected antibody specificity. This approach has been described as a practical alternative in settings where molecular testing is not readily available.

Several case series, including two P‐null cases from Western India and additional donors and family clusters, confirm the autosomal‐recessive inheritance and highlight the challenge of sourcing compatible units [4]. The Middle East has been specifically mentioned as a region where the P phenotype is encountered, supporting the relevance of our finding in an Iranian patient. To our knowledge, the P‐null phenotype remains rarely reported from Iran in the context of transfusion medicine, which gives this case added regional importance.

### 5.1 Obstetric Implications and Anti‐PP1Pk–Associated Pregnancy Loss

Even though our patient has had one live birth without a reported miscarriage, the presence of anti‐PP1Pk carries significant implications should she become pregnant again. Multiple reports associate maternal P‐null status and anti‐PP1Pk with recurrent early pregnancy loss and, less commonly, with late fetal death or HDFN [12]. The pathophysiology differs somewhat from classical RhD alloimmunization: P antigens are highly expressed on trophoblast and placental tissues, and maternal anti‐PP1Pk can damage the placenta from very early gestation, even before significant fetal erythropoiesis, leading to implantation failure or early miscarriage rather than primarily fetal anemia [5].

An Iranian series of women with recurrent pregnancy loss and anti‐P/anti‐PP1Pk antibodies reported successful pregnancies after intensive therapeutic plasma exchange, supporting an immune‐mediated mechanism and the potential reversibility of this condition with appropriate therapy. More recent case reports from other countries have demonstrated successful pregnancies in P‐null women using various combinations of serial plasma exchange, high‐dose IVIG, close ultrasound surveillance, and intrauterine transfusion when needed [6, 12]. These data suggest that, although the risk is substantial, proactive multidisciplinary management can permit favorable fetal outcomes.

### 5.2 Transfusion Management and Patient Blood Management (PBM)

From a transfusion perspective, our case reinforces general principles for managing antibodies against high‐prevalence antigens [4, 5, 13]. Once a P‐null phenotype with anti‐PP1Pk is suspected, it is essential to:

1. Confirm the specificity through extended serologic workup in collaboration with a reference or immunohematology laboratory.
2. Flag the patient permanently in transfusion service databases and, where available, in national hemovigilance systems as a P‐null individual.
3. Register the patient with rare donor programs and attempt to identify phenotypically matched donors, including targeted family testing in selected cases.
4. For any future transfusion need, restrict support to ABO/Rh‐compatible P‐null red cells or, if absolutely unavoidable in life‐threatening situations, carefully selected least‐incompatible units with intensive monitoring for hemolysis.

Our patient benefited from early recognition of the antibody and a clear laboratory recommendation that only P‐null units should be used if transfusion becomes necessary. In the context of elective surgery, the identification of such a phenotype should prompt a PBM strategy, including optimization of preoperative hemoglobin (iron therapy ± erythropoiesis‐stimulating agents when appropriate), meticulous surgical hemostasis, use of cell salvage where feasible, and restrictive transfusion thresholds to minimize exposure to incompatible cells [1].

In Iran, a national rare blood group donor program has been established by the IBTO. This program includes extended red blood cell phenotyping of selected donors and maintenance of a rare blood group registry, which plays a crucial role in providing compatible blood units for patients with rare phenotypes and antibodies, such as those described in the present case.

### 5.3 Clinical Recommendations and Implications

Based on the available literature and the experience from this case, several practical recommendations can be made:

Routine antibody screening remains indispensable even in apparently low‐risk elective surgery. A pan‐reactive pattern with negative autocontrol should trigger suspicion of an antibody to a high‐frequency antigen, including anti‐PP1Pk, and mandate referral to a specialized laboratory.

Transfusion services, particularly in regions such as the Middle East and Iran, should be aware that the p phenotype, although rare, is present in their populations. Education of blood bank and clinical staff about the P1PK system and anti‐PP1Pk can reduce delays in diagnosis and improve coordination with national or international rare donor registries.

Molecular testing, while not performed in our case, is increasingly accessible and can confirm the underlying A4GALT variant. This information can be valuable for family counseling and for contributing to global databases of rare blood group alleles.

In conclusion, this case underscores the critical importance of routine preoperative antibody screening in identifying rare but clinically significant immune‐hematologic phenotypes such as the P‐null type with probable anti‐PP1Pk. Early recognition in our patient—made before any transfusion exposure—allowed appropriate risk stratification, avoidance of incompatible transfusion, and the establishment of long‐term precautions for future surgical or obstetric care. Given the high potency of anti‐PP1Pk and its association with severe hemolytic transfusion reactions and pregnancy loss, accurate documentation, patient counseling, and coordination with reference laboratories and rare‐donor registries are essential. This report contributes valuable regional insight into a phenotype rarely described in Iran and highlights the need for heightened clinical awareness and systematic planning to ensure optimal safety for affected individuals.

## 6 Limitation

The absence of direct molecular testing represents a limitation of this report; however, indirect serologic–molecular correlation provides supportive evidence for the diagnosis.

## Author Contributions

**Alireza Abdollahi:** conceptualization, methodology, project administration, supervision, writing – original draft. **Aysan Nozheh:** investigation, resources, validation, writing – original draft, writing – review and editing. **Hassan Sohrabinia:** data curation, resources, validation, writing – review and editing. **Samaneh Salarvand:** data curation, formal analysis, investigation, software, writing – original draft.

## Funding

The authors have nothing to report.

## Ethics Statement

This study was conducted in accordance with the ethics principles outlined in the Declaration of Helsinki. Ethics approval was obtained from the ethics committee of Tehran University of Medical Sciences.

## Consent

Written informed consent was obtained from the patient for publication of this case report and any accompanying images.

## Conflicts of Interest

The authors declare no conflicts of interest.

## Data Availability

All data generated or analyzed during this study are included in this published article.
